# Race and ethnicity data for first, middle, and surnames

**DOI:** 10.1038/s41597-023-02202-2

**Published:** 2023-05-19

**Authors:** Evan T. R. Rosenman, Santiago Olivella, Kosuke Imai

**Affiliations:** 1 Department of Mathematical Sciences, Claremont McKenna College (incoming), Claremont, USA; 2 Harvard Data Science Initiative, Harvard University, Cambridge, USA; 3 Department of Political Science, University of North Carolina at Chapel Hill, Chapel Hill, USA; 4 Department of Government, Harvard University, Cambridge, USA; 5 Department of Statistics, Harvard University, Cambridge, USA

**Keywords:** Politics, Interdisciplinary studies

## Abstract

We provide the largest compiled publicly available dictionaries of first, middle, and surnames for the purpose of imputing race and ethnicity using, for example, Bayesian Improved Surname Geocoding (BISG). The dictionaries are based on the voter files of six U.S. Southern States that collect self-reported racial data upon voter registration. Our data cover the racial make-up of a larger set of names than any comparable dataset, containing 136 thousand first names, 125 thousand middle names, and 338 thousand surnames. Individuals are categorized into five mutually exclusive racial and ethnic groups — White, Black, Hispanic, Asian, and Other — and racial/ethnic probabilities by name are provided for every name in each dictionary. We provide both probabilities of the form ℙ(race|name) and ℙ(name|race), and conditions under which they can be assumed to be representative of a given target population. These conditional probabilities can then be deployed for imputation in a data analytic task for which self-reported racial and ethnic data is not available.

## Background & Summary

In the absence of self-reported data, researchers frequently seek to predict the race and ethnicity of individuals. Such prediction tasks are essential for identifying racial disparities in public health and social sciences. Because researchers often have access to information about individuals’ names and addresses, they typically utilize these data to make informed predictions about individual race. A leading methodology is Bayesian Improved Surname Geocoding (BISG), which uses Bayes’ rule to obtain predictions about an individual’s race by combining Census-derived race-name distributions with the decennial Census counts for certain geographical units such as Census blocks and tracts^1–5^. BISG has been deployed in studies on racial disparities in lending^6^, policing^7^, eviction^8^, voter turnout^9^, and many other application areas.

The validity of BISG and similar methodologies is, however, reliant on accurate estimates of the race-name relationship, which are operationalized as either the conditional probabilities of race given name or the conditional probabilities of name given race. A commonly-used BISG implementation, included in the R package wru^10^, combines two U.S. Census datasets to estimate these distributions for surnames. The first is the Census Bureau’s surname list, which provides the racial distribution of surnames appearing at least 100 times. The 2010 version of this list contains about 160,000 names, covering approximately 90% of the U.S. population^4^. The second source is the Census’s Spanish surname list, which contains roughly 12,000 common Hispanic surnames, approximately half of which are not in the Census surname list. Despite their overall coverage, these lists can still fail to contain the surnames of disproportionately large subsets of minority populations — especially Asian Americans^11^. Other resources for race-name distributions^12^ suffer from similar challenges in terms of differential coverage across racial groups.

Our new dataset, which is made publicly available in the Harvard Dataverse^13^, substantially expands on the available data for estimating race-name distributions. We make use of voter files from six states in the American South which collect data about voters’ self-reported race and ethnicity. The six states are: Alabama, Florida, Georgia, Louisiana, North Carolina, and South Carolina. The voter files — a compendium of all registered voters in a given state at a given time — are sourced from L2, Inc., a leading national non-partisan firm and the oldest organization in the United States that supplies voter data and related technology to candidates, political parties, pollsters, and consultants for use in campaigns. For improved sample sizes and enhanced privacy, we aggregate seven voter files per state, dated between 2018 and 2021.

As of early 2021, these states comprised nearly 38 million total registered voters. More than 90% of voters provide self-reported race information — data that is widely accepted as the gold-standard definition of an individual’s race^12^. These datasets allow us to compile rich dictionaries mapping first, middle, and surnames to their empirical frequencies by race. The first name dictionary contains 136,000 unique first names; the middle name dictionary contains 125,000 unique middle names; and the surname dictionary contains 338,000 unique surnames. For each type of name, we provide a dataset of probabilities of the form ℙ(race|name) and ℙ(name|race). To better protect the privacy of individuals, these datasets omit names that appear fewer than 25 times across all voter files.

In a separate paper^11^, we demonstrate the practical utility of this data enhancement, which is now incorporated into the latest version of wru. We analyzed the voter file data from the aforementioned Southern States, using a leave-one-state-out approach to show that the additional data improves the out-of-sample predictive accuracy of BISG while maintaining good calibration of predicted probabilities. Specifically, by assigning each individual to the maximum *a posteriori* race class based on the standard BISG procedure, we achieved an overall misclassification rate of 13.2%. This represented a significant improvement over the error rate of 16.7% achieved using only the surname data sourced from the Census (see Table 5 in the aforementioned paper). These improvements were particularly substantial for racial minorities (e.g., false negative rates were reduced from 50% to 41% among Asian Americans, as discussed in more detail in the prior paper).

Although these empirical results suggest its potential benefits, our data set is not without limitations. In particular, it is important to understand the conditions under which data sourced from voter files can be used to study a certain target population, such as the entire United States (U.S.). In the section titled “Potential Discrepancies: Voter files vs. United States Population”, we state these conditions explicitly and formally. In the “Technical Validation” section, we evaluate the empirical validity of these conditions for our surname data by comparing the race-surname relations derived from our data against those based on the Census data, using the surnames that appear in both data sets. We find that these probabilities correlate highly, suggesting that the required assumptions may hold approximately for surnames. Unfortunately, given the lack of corresponding Census data for first and middle names, it is not possible to conduct a similar exercise to assess the empirical validity of our stated conditions for first and middle names. Instead, we compare the conditional probabilities based on our first name data against those based on mortgage applications^12^, which is the only publicly available data set containing the racial make-up of U.S. first names. While the comparison reveals important differences between these two data sets, further research is needed to determine whether either data set provides name-race relations approximately representative of the U.S. population.

## Methods

### Voter files

As mentioned above, voter files for all six U.S. Southern states were sourced from L2, Inc. L2 cleans and maintains voter files obtained from states. This process includes the removal of voters whom they consider as inactive, potentially contributing to a lack of representativeness. We clarify the assumptions required for the use of voter files in BISG and examine their validation later in this section and in the “Technical Validation” section.

We included seven voter files per state, dating between 2018 and 2021. Aggregation over multiple voter files was achieved by simply concatenating the files. Hence, individuals registered to vote over the entire period between 2018 and 2021 are counted seven times, while those entering or exiting the file are counted fewer times.

Table 1 summarizes the data sourced from each state. For ease of interpretation, we provide the average number of voters from each state across the seven voter files, as well as the average racial composition. Self-reported racial data was listed on the file for the overwhelming majority of voters across states. For Florida, Georgia, and North Carolina, approximately 3.3%, 9.3%, and 5.6% of registered voters do not have self-reported race.

**Table 1:** Aggregate racial distributions the voter files in each state.

State	Total # of Reg Voters (Million)	% White	% Black	% Hispanic	% Asian	% Other	% No Race
Alabama	3.1	70.0	26.8	1.0	0.6	1.1	0.5
Florida	13.0	62.1	13.6	17.1	2.0	2.0	3.3
Georgia	6.3	53.6	29.9	3.2	2.4	1.7	9.3
Louisiana	2.8	63.5	31.3	1.4	0.9	2.9	0.0
North Carolina	6.1	66.9	21.1	2.8	1.3	2.2	5.6
South Carolina	3.0	69.1	27.3	1.7	1.0	0.9	0.1
Total	34.3	62.8	21.8	7.9	1.6	1.9	4.0

We provide the average over the seven voter files sourced for each state, dating from 2018 to 2021. Percentages may not add up to precisely 100% due to rounding.

While every Southern state is majority White, each has a large Black population, ranging from 14% to 31% of the total population of registered voters in the state. There are relatively fewer Hispanic voters in the South, with such voters comprising a large proportion of the population in Florida (17%), but less than 5% in all other states. The Asian proportion of the population is even smaller, ranging from less than 0.6% of the population (Alabama) to 2.4% (Georgia). Nevertheless, even for racial groups that comprise a small proportion of the population, these files provide full name and self-reported racial data for hundreds of thousands of voters.

### Data processing

Data processing for first and middle names was minimal. For each name type, name tallies by race were computed by first removing punctuation marks and intervening white spaces, and iterating through each file. Punctuation and space removal were achieved using the gsub() function in R, replacing the standard regular expressions [:punct:] and [:blank:] with an empty string. Voters were categorized into five racial groups — White, Black, Hispanic, Asian, and Other — based on self-reported racial data. Each of these categories maps directly to categories reported on the L2-sourced voter file, with the exception of the Other category, which is an amalgam of the Native American and Other categories on the file.

Note that the L2-sourced file incorporates some post-processing on top of the base voter file. While states often collect race and ethnicity data separately — such that individuals may identify as, e.g., both Black and Hispanic — L2 collapses these categorizations into a single field. Using a standard convention^12^, Hispanic voters are grouped into a single category regardless of race, while non-Hispanic voters are identified with their self-reported racial category.

Once the name tallies were obtained, names were cast to uppercase, and converted to UTF-8 encoding using the iconv() function in R. A small number of names that contained non-ASCII characters were removed from the first name and surname dictionaries. Such names were collapsed together with an empty string category corresponding to missing middle names for the middle name dictionary. Missingness is rather common for middle names, as many individuals have only a first name and a surname, so the empty string is included as an entry in the middle name dictionary. After processing, the name tallies table was normalized row-wise to generate a table of probabilities ℙ(race|name), as well as column-wise to generate a table of probabilities ℙ(name|race).

For privacy protection purposes, we filtered out any names that appeared fewer than 25 times in our dataset (i.e., across the six states and seven voter files per state). All names appearing fewer than 25 times are collapsed into a single token, “ALL OTHER NAMES,” with the appropriate probabilities reported for each dictionary. After processing and filtering, the final dictionaries provide rich details about race-name distributions. Our first name dictionaries contain roughly 136,000 unique names, while our middle name dictionaries contain 125,000 unique names and our surname dictionaries contain 338,000 unique names. A summary of the frequency of different names in each dictionary can be found in Table 2.

**Table 2:** Sample sizes by name for names in the first name, middle name, and surname dictionaries.

Sample Size Range	First Names		Middle Names		Surnames	
	Count	Frequency	Count	Frequency	Count	Frequency
[25, 49]	58,112	42.8%	54,009	43.1%	136,989	40.5%
[50, 99]	31,932	23.5%	29,147	23.3%	86,004	25.4%
[100, 249]	22,853	16.8%	20,562	16.4%	60,441	17.9%
[250, 499]	9,244	6.8%	8,564	6.8%	22,972	6.8%
[500, 999]	5,469	4.0%	5,233	4.2%	13,101	3.9%
1000+	8,166	6.0%	7,740	6.2%	18,661	5.5%
Total	135,776	100.0%	125,255	100.0%	338,168	100.0%

Sample sizes are computed over seven voter files per state for each of Alabama, Florida, Georgia, Louisiana, North Carolina, and South Carolina. The minimum number of observations for each name is 25.

### Potential discrepancies: voter files vs. U.S. population

Our goal is to provide a dataset that reasonably captures name-race distributions for the United States population. However, our data incorporates two potential sources of bias. First, our data is drawn only from the population of registered voters. Aside from the standard restrictions that voters be U.S. citizens and over the age of 18, there are considerable demographic differences between the populations of registered voters and nonvoters^14^. The second source of bias is geographic: our data is drawn from only six states, whose demographics differ from those of the entire country. We can quantify the discrepancies between our sample and the broader American population on the measure of race and ethnicity by looking at the final row of Table 1 above, which gives the marginal distribution across the voter files. While the proportion of White Americans in our sample roughly matches the national population, Hispanic and Asian Americans are severely underrepresented, and Black Americans are modestly overrepresented^15^.

We consider the conditions under which each of these two biases would *not* affect the accuracy of subsequent racial prediction via BISG (or a similar technique) among people who are not part of our sample. Regarding the first source of bias, it can be shown that under the following assumption, the conditional distribution of race given name, ℙ(race|name), is identical between registered voters and non-registered voters.

#### Assumption 1.a

*Conditional on name n*_*i*_*, the individual’s race r*_*i*_ *and voter registration status reg*_*i*_ *are independent. That is*,

$${r}_{i}\perp \,\perp {reg}_{i}\,| \,{n}_{i}.$$

This assumption is violated if, for example, Black and White voters with an identical surname have different propensities to register to vote. Alternatively, our data can also be used to obtain the conditional probabilities of name given race, ℙ(name|race), if the following assumption holds:

#### Assumption 1.b

*Conditional on the race r*_*i*_ *of an individual i, the individual’s name n*_*i*_ *and voter registration status reg*_*i*_ *are independent. That is*,

$${n}_{i}\perp \,\perp {reg}_{i}\,| \,{r}_{i}.$$

The assumption is violated, for example, if Black voters with different surnames have varying propensities to register. If either Assumption 1.a or 1.b is satisfied, researchers can reliably use our data in combination with the Census name data to obtain relevant conditional probabilities through one of the BISG formulae.

Similarly, for the second source of bias, what matters is whether the conditional (rather than marginal) probabilities match those derived from the target national population. In particular, the relevant conditional probabilities remain valid so long as each of the following two assumptions holds,

#### Assumption 2.a

*Conditional on name n*_*i*_*, the individual’s race r*_*i*_ *and voter geolocation geo*_*i*_ *are independent. That is*,

$${r}_{i}\perp \,\perp {geo}_{i}\,| \,{n}_{i}.$$

#### Assumption 2.b

*Conditional on the race r*_*i*_ *of an individual i, the individual’s name n*_*i*_ *and geolocation geo*_*i*_ *are independent*.

$${n}_{i}\perp \,\perp {geo}_{i}\,| \,{r}_{i}.$$

Assumption 2.a is violated if, for instance, two Hispanic and Black voters who share the same surname are systematically inclined to live in different places. In turn, Assumption 2.b would fail to hold if, for example, knowing the first name of two White voters could help us predict their place of residence.

Although it is impossible to fully verify these assumptions, we can evaluate their credibility by comparing conditional distributions derived from our data to those derived from other sources that have broader geographic and domain diversity (see the “Technical Validation” section below).

## Data Records

The data is available for download in the Harvard Dataverse^13^. All data are stored in comma-separated values (CSV) format, for accessibility. We also provide each of these in RData format, which can be accessed by downloading the files locally and reading them into R using the standard load() function. Six files are available: first_nameRaceProbs, middle_nameRaceProbs, and last_nameRaceProbs (each in CSV and RData formats), which provide conditional probabilities ℙ(race|name); as well as first_raceNameProbs, middle_raceNameProbs, and last_raceNameProbs (again, in our two formats), which contain conditional probabilities ℙ(name|race). Regardless of the file format, datasets all have six columns. The first column in each dataset gives the name, which has been cast to uppercase. The next five columns give the appropriate conditional probabilities for White, Black, Hispanic, Asian, and Other individuals derived from the Southern voter files.

## Technical Validation

In this section, we first compare the relevant conditional probabilities based on our surname data against those based on the U.S. Census surname data. This allows us to examine the validity of the above assumptions that are required when using our data for race imputation in a national setting via a modeling technique such as BISG. We also compare these conditional probabilities based on our first name data against those derived from mortgage applications in the entire United States. Unlike the Census data, the mortgage application data are not representative of the population. Therefore, although we show the difference between the two data sets, this second comparison does not provide useful information about the validity of the required assumptions, and we provide it simply for reference.

### Comparison against Census surname list

We first link our surname dictionary to the 2010 Census surname list and compare the conditional probabilities ℙ(race = *r*|surname) and ℙ(surname|race = *r*) for each *r* ∈ {White, Black, Hispanic, Asian, Other} and for every one of the linked names. The 2010 version of the surname list is used because the 2020 Census surname list has not yet been released, as of the time of writing (viz., early 2023). Of the 162,253 names on the 2010 Census surname list — representing all names that appeared more than 100 times in that Census — we are able to match about 89% of them to entries in our surname dictionary. The rate would be significantly higher — 97% — if we include all names that appear in voter files. As noted earlier, our privacy-protecting restriction excludes names appearing fewer than 25 times across the voter files. Nonetheless, the names linked across the Census surname list and our privacy-protected surname dataset account for more than 91% of the the total voter file records across the six states and seven voter files per state.

**Fig. 1:**
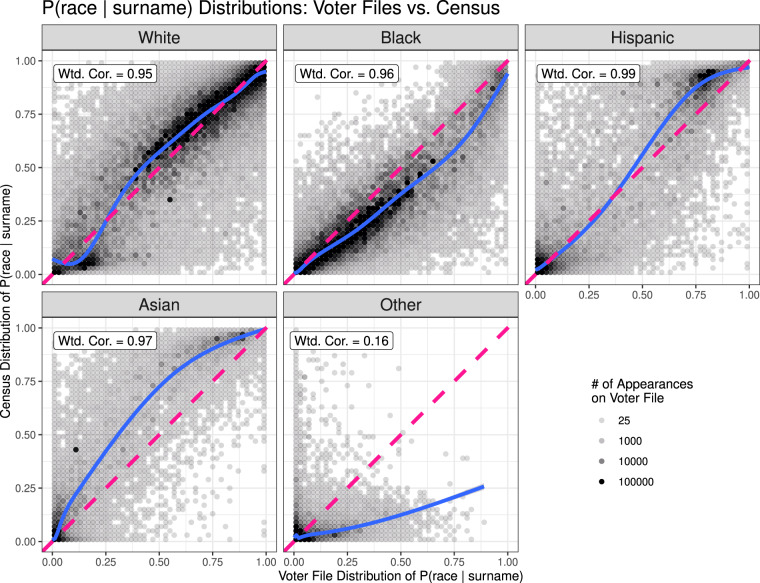
ℙ(race = *r*|surname) values for each racial and ethnic group *r*, computed for linked names via the Census (*y* axis) and via the voter files (*x* axis). Data is coarsened such that each dot represents 2% of the range along each axis. The dots’ opacity corresponds to the total number of voter file appearances for names that fall into each range. For comparison, the unweighted correlations are 0.92, 0.93, 0.91, 0.93, and 0.26, respectively.

**Fig. 2:**
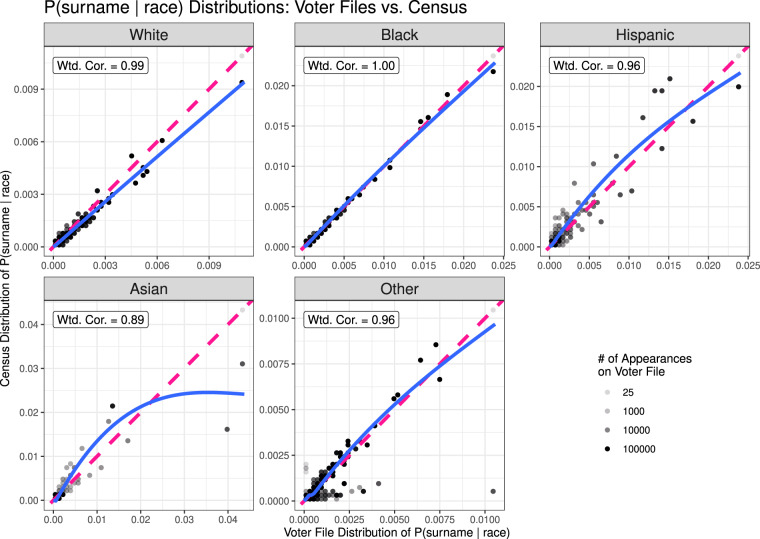
ℙ(surname|race = *r*) values for each racial and ethnic group *r*, computed for linked names via the Census (*y* axis) and via the voter files (*x* axis). Data is coarsened such that each dot represents 2% of the range along each axis. The dots’ opacity corresponds to the total number of voter file appearances for names that fall into each range. For comparison, the unweighted correlations are 0.98, 1.00, 0.95, 0.89, and 0.84, respectively.

Figures 1, 2 plot the comparisons. In Fig. 1, the Census distribution of ℙ(race = *r*|surname) is given on the *y*-axis, whereas the voter file distribution of the probabilities is given on the *x*-axis. The same holds for ℙ(surname|race = *r*) in Fig. 2. For ease of visualization, we plot a coarsened version of the data, where the the opacity of each dot reflects the total number of voter file appearances of surnames that fall into that region of the plot (a darker color means a greater number of voters). The weighted correlations reported on each plot are computed using all the data, with weights also corresponding to the frequency with which such names appear on the voter files. In solid blue, we plot lowess curves obtained via a generalized additive model, which are also weighted by the number of appearances of a surname on the voter file. In dashed pink, we plot the 45-degree line, which would indicate perfect agreement between the two sets of conditional probabilities.

Figure 1 simultaneously assesses the plausibility of Assumptions 1.a and 2.a. Weighted correlations are 95% or higher for all racial and ethnic groups except for the “Other” category. This indicates that there is general concordance between the Census data and the voter file data. That is, surnames that the Census finds to be strongly indicative of membership in a particular racial group are typically indicative of the same group membership in the voter file data. These high correlations provide evidence for the approximate validity of Assumptions 1.a and 2.a. The sole group for which concordance is not observed is the “Other” group, for which the weighted correlation falls to a much less impressive 16%. This is not surprising because the “Other” category is quite small and an amalgam of several smaller racial and ethnic groups. Further discussion of these discrepancies can be found at the end of this section.

Moreover, the local regression curves of best fit (plotted in solid blue) adhere reasonably closely to the 45-degree line (in dashed pink), indicating that while there may be some deviation from the Census probabilities, there is little systematic bias. *Conditional on surname*, there is almost no evidence that White and Hispanic individuals are over- or underrepresented in the voter files relative to the Census. We do find that, conditional on surname, Black voters are slightly over-represented, and Asian voters are under-represented, in the voter files relative to the Census. Because these discrepancies mirror the relative over- and under-representations of these racial groups in the South relative to the United States, these trends likely indicate minor violations of Assumption 2.a. However, they could also partially be attributable to violations of Assumption 1.a, or to the temporal discrepancy when using 2010 Census probabilities to analyze 2020 voter files.

Figure 2 compares the conditional probabilities of surname given race between our data and the 2010 Census name list, and hence simultaneously asseses the plausibility of Assumptions 1.b and 2.b. For White, Black, Hispanic, and Other individuals, there exists high correlation between the probabilities computed on the two datasets, and no evidence of systematic bias is found. For Asian Americans, the weighted correlation is still nearly 0.90, but it is notably lower than the other racial groups. Two outliers are visible on the righthand side of the plot, corresponding to two popular surnames — “Nguyen” and “Patel” — which drive down the correlation. These two names, which are common Vietnamese and Indian surnames, respectively, are more common among Asian individuals on the voter files than in the Census. This is almost certainly due to the fact that Vietnamese-Americans and Indian-Americans comprise a larger proportion of Asian Americans in the South than in the country at large^16,17^, inducing a modest violation of Assumption 2.b. We note that these data may not perform optimally when making predictions on Asian populations whose distribution of national origin differs markedly from that of Southern states. But otherwise, we find that Assumptions 1.b and 2.b hold reasonably well in these data.

This validation study does not test the plausibility of assumptions among the names in our surname dictionary that we are unable to link to the Census. The results, however, show that for the overwhelming majority of our voter file population — those whose names can be linked to the Census surname list — the required assumptions appear plausible.

We conclude this section with a brief discussion of name discrepancies between the Census and voter files for the Other category. In Table 3, we investigate the ten most common names such that ℙ(race = “Other”|name) differs by more than 25 percentage points between the Census and the voter files. We find two clear trends. At least four of the names — “Chavis”, “Locklear”, “Oxendine”, and “Brayboy” — are traditional Native American surnames associated with the Lumbee tribe in North Carolina^18^. Individuals with these surnames are frequently categorized by the Census in the Other category, while they are overwhelmingly categorized as either White or Black on the voter file. Additionally, at least three of the names — “Persaud”, “Persad”, and “Baksh” — are recognizably of South Asian or Indo-Caribbean origin. Among these names, the Census typically categorizes more individuals as Asian, while the voter file data more typically categorizes these individuals as Other. This may explain the relatively low concordance seen between the datasets for this group only.

**Table 3:** Ten most common names in the voter files such that the estimate of ℙ(race = “Other”|name) differs by more than 25 percentage points between the Census surname list and the voter file estimate.

Surname	Census Probabilities					Voter File Probabilities					
	White	Black	Hispanic	Asian	Other	White	Black	Hispanic	Asian	Other	Avg. # of Voter File Appearances
CHAVIS	28	40	4	0	28	46	51	1	0	2	3,694
LOCKLEAR	19	3	1	0	77	80	12	1	2	4	1,959
PERSAUD	10	21	5	52	13	8	8	6	36	42	1,509
OXENDINE	22	8	1	0	69	67	26	1	2	4	937
BILLIOT	46	2	2	0	50	90	2	0	1	7	925
JOE	12	25	3	23	37	8	79	0	10	4	694
MCGIRT	24	47	3	0	26	39	60	1	1	0	490
BRAYBOY	7	58	1	0	32	13	84	1	0	2	433
PERSAD	10	35	5	35	15	8	8	5	34	43	294
BAKSH	9	26	5	48	12	7	9	3	31	49	276

We observe that many of the names appear to be of Native American, South Asian, or Indo-Caribbean origin.

### Comparison against the first-name dataset from mortgage applications

We also compare our data against the database of first-name racial distributions compiled by Tzioumis^12^. These data were created by merging three proprietary mortgage application datasets, drawn from applications submitted between 2007 and 2010. The lenders comply with requirements under the Home Mortgage Disclosure Act (HMDA), meaning that they collect self-reported race and ethnicity data about the applicants. The total sample size of first name observations is about 2.66 million, which is smaller than our voter file data. After some careful processing steps, Tzioumis obtains a list of 4,250 first names along with their corresponding race and ethnicity distributions.

As mentioned above, the mortgage data set is not representative of the population. As such, the comparison is not informative about the validity of the assumptions that are required for the use of our data set to make race predictions at the national level. Unfortunately, given the lack of Census first and middle name lists, we cannot directly verify the national representativeness of our first and middle name lists. Nor can we examine the appropriateness of Tzioumis’ first name list, for the same reason. Thus, our comparison here is confined to the simple description of the differences between the two first-name data sets.

Despite this limitation, we believe that this comparison provides useful information for a couple of reasons. First, as far as we are aware, Tzioumis’ is the sole database of first-name racial distributions that is publicly available in the United States. Second, Tzioumis directly cautions against using voter file data for the purposes of race imputation, writing:

*In terms of voter registration data, the vast majority of states either do not have information on the race and ethnicity of the voters, or have restrictions on the use of the data, or both. Moreover, in terms of data integrity when combining data across states, there may be concerns as data maintenance and voter purge practices vary substantially across election boards.*

We echo Tzioumis in noting that the states which provide race data on their voter files often have different demographics than the broader U.S. population. However, our analysis in the prior section indicates that, once we focus on *conditional* distributions, the magnitude of this bias shrinks considerably for surname-race distributions.

Of the 4,250 unique first names on the mortgage application list, we are able to link all but two of them (“Hakop” and “Vahik”) to entries in our first name dataset. The linked entries represent just 3.1% of the total names in our dataset, reflecting the fact that our dictionary is significantly larger than the mortgage names dictionary. Nonetheless, the linked entries cover 85.6% of the records in our voter files from individuals who report their race. This owes to the concentration of first names: the vast majority of individuals have a popular first name, while concentration is considerably lower for surnames^12^.

The racial and ethnic categorization of the mortgage application data is quite similar to ours. In particular, self-identified Hispanics are unified into a single group, regardless of their listed race, while the White, Black, and Asian groups are defined as non-Hispanic members of those racial groups. Tzioumis provides two additional categories — American Indian or Alaska Native (0.17% of observations) and Multi-race (0.16%) — that are not present in our categorization. For the purposes of comparison, we aggregate these two groups and compare them against our “Other” group (which comprises about 2% of our sample) as done above.

**Fig. 3:**
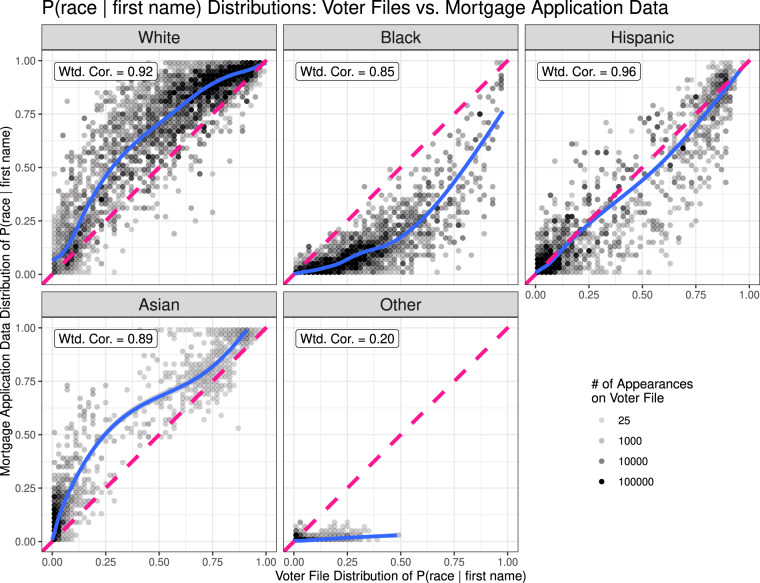
ℙ(race = *r*|first name) values for each racial and ethnic group *r*, computed for linked names via the Tzioumis mortgage application data (*y* axis) and via the voter files (*x* axis). Data is coarsened such that each dot represents 2% of the range along each axis. The dots’ opacity corresponds to the total number of voter file appearances for names that fall into each range.

In Fig. 3, we plot the comparisons for ℙ(race|first name), using an identical set-up to the Census comparison. The mortgage application distribution of ℙ(race = *r*|first name) is given on the *y*-axis and the voter file distribution of the probabilities is given on the *x*-axis. We again report weighted correlations and provide a lowess curve of best fit in solid blue.

The comparison is instructive in a number of ways. Although weighted correlations are reasonably high for all groups except the “Other” category, they are lower than in comparison with the Census surname list. Moreover, there appear to be somewhat pronounced systematic discrepancies between the racial distributions conditional on the name. The mortgage application data appears to assign considerably higher probabilities of being White or Asian than the voter file data, and considerably lower probabilities of being Black, after conditioning on first name.

Aggregating the Tzioumis data, we find that the sample is 82.3% White, 4.2% Black, 6.9% Hispanic, 6.3% Asian, and 0.3% “Other”. These marginals are not entirely unexpected: a well-established finding suggests that Whites are significantly more likely than members of any other racial or ethnic group to own their own homes^19^. It is hence no surprise that mortgage applications skew disproportionately toward White applicants.

Recall that the voter file data does not appear to be systematically Whiter than the U.S. population, conditional on surname. This implies that the differences in White probabilities are likely to be driven by overrepresentation of White applicants in the mortgage application data. Baselining against the Census results, it appears that that our data may tend to assign slightly too high probabilities of being Black conditional on first name, while the Tzioumis data may tend to assign *too low* probabilities. There does not appear to be a systematic difference among Hispanic first names. The results are less clear for Asian Americans, but the Tzioumis data may provide more accurate national probabilities for this group due to better representation among mortgage applicants.

The “Other” categorization is, in aggregate, nearly 10 times less frequent among the mortgage application data than in the voter file data. The poor correlation exhibited between the datasets on ℙ(race = “Other”|first name) is largely driven by this discrepancy in marginal composition.

**Fig. 4:**
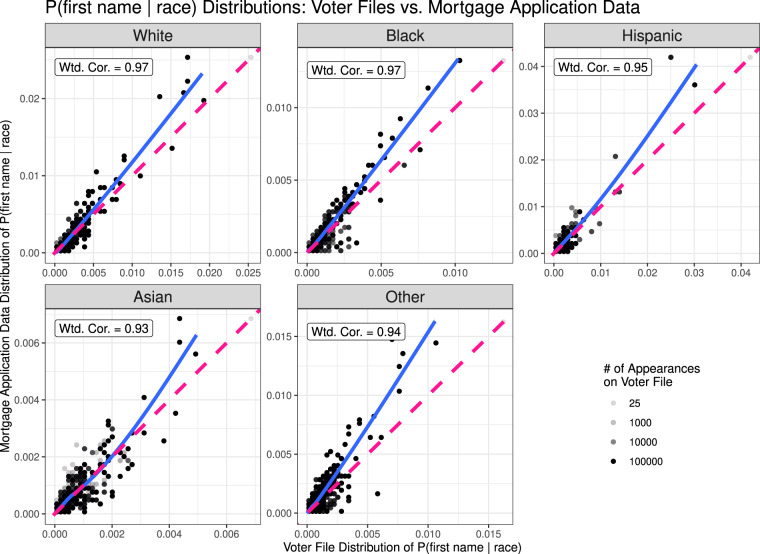
ℙ(first name|race = *r*) values for each racial and ethnic group *r*, computed for linked names via the Tzioumis mortgage application data (*y* axis) and via the voter files (*x* axis). Data is coarsened such that each dot represents 2% of the range of each axis. The dots’ opacity corresponds to the total number of voter file appearances for names that fall into each range.

In Fig. 4, we plot the comparisons for the inverted conditional probabilities, ℙ(first name|race). Here, we observe high correlations for all racial groups (even the “Other” group) for name probabilities conditional on race. One notable discrepancy, however, is that the best fit curve (in solid blue) arcs above the 45-degree line in all cases. This owes to the fact that the mortgage application data shows higher first name concentration within each racial group than the voter file data. For example, the mortgage data shows that 2.6% of Whites are named “Michael” while our data indicates that only 1.7% of Whites are named “Michael”. The discrepancy may be driven by a complex interplay with the gender distribution of the datasets.

The voter file data is slightly biased toward women, with 53.7% of the registered individuals identifying as female vs. 50.5% for the population at large^15^. Yet the mortgage application data appears to be biased to a greater degree, and in the opposite direction. Using the R package gender^20^, we are able to estimate gender probabilities for 97% of the names in the mortgage dataset, from which we estimate that approximately 44.2% of the individuals in the underlying dataset are female. Because there is greater concentration among male first names (that is, a higher proportion of men are given popular male first names than women are given popular female first names), the bias toward men in the mortgage data may induce greater name concentration overall^21^.

## Usage Notes

We provide a new dataset to estimate race-name distributions for first, middle, and surnames, derived from publicly available voter files in six Southern states. These dictionaries are a rich and novel resource. The surname dictionary contains twice as many names as the the Census surname list, the best public resource for estimating surname-race distributions. The first name dictionary contains more than 30 times as many names as the Tzioumis mortgage application dataset, which is the only data set of the racial make-up of first names currently available in the United States. The middle name dictionary is, to our knowledge, the first such dictionary of its kind.

Having access to these dictionaries means that, when the assumptions laid out in section “Potential Discrepancies” hold, race imputation tasks can be conducted on a wider subset of the population, allowing public policy researchers to more credibly evaluate disparate racial impacts. In a related paper^11^, we show that having access to all three name-race distributions (as opposed to surname-only, or surname and first name only dictionaries) can improve race prediction accuracy substantially — especially when using the data in a fully Bayesian framework to classify racial minorities.

While our analysis shows that the dictionaries are reasonably representative of national race distributions, they are not drawn from a random sample of the U.S. population. Registered voters must be over 18 and must be U.S. citizens, and they also typically differ from non-voters in terms of socioeconomic status. Moreover, our data is drawn from Southern states that have larger Black populations, but smaller Asian and Hispanic populations, than the country as a whole. Researchers should carefully consider the populations on which they would like to deploy these data, and the conditions under which such deployment could lead to erroneous conclusions (see our discussion above).

When considering surnames, it will normally make sense to use our dictionary to supplement — rather than supplant — the Census surname list, such that national race distributions are used when available. This is the approach taken in our accompanying paper^11^. Overall, further research is needed to establish the national representativeness of available name-race distributions.

The final usage note concerns the definition of the “Other” category. The racial categorization provided with these data is appropriate for many race imputation tasks, but may be problematic if researchers are interested in finer categorizations of individuals. In particular, these dictionaries may not be useful for identifying Native American communities, and hence should be used with caution in states with large Native American populations, such as Alaska and Oklahoma.

## Data Availability

The underlying voter files are proprietary and sourced from L2, Inc. The data can be directly purchased from L2, Inc. Otherwise, the count files generated herein can be recreated by acquiring voter files from each state. Among the six states we have considered, data from North Carolina is the most straightforward to obtain, as the voter file can be downloaded for free from the State Board of Elections website. Data processing code is straightforward and involves iterating through each voter file and tallying names by race. Initial processing code was written in Python (version 3.6), while a small amount of post-processing was done in R (version 4.1.2). These steps are described in the *Data Processing* subsection. Sample data processing code are available alongside the count files in the Harvard Dataverse^13^. Code to recreate each of the figures can be found in a separate repository in the Harvard Dataverse^22^.
